# Feasibility and safety of 0.6% sodium alginate in endoscopic submucosal dissection for colorectal neoplastic lesion: A pilot study

**DOI:** 10.1002/deo2.313

**Published:** 2023-11-04

**Authors:** Hajime Nakamura, Rie Morita, Ryo Ito, Akira Sakurada, Natsumi Tomita, Yuya Hirata, Yusuke Kanari, Yuya Komatsu, Kunihiro Takanashi, Tomonori Anbo, Shinichi Katsuki

**Affiliations:** ^1^ Department of Gastroenterology Otaru Ekisaikai Hospital Hokkaido Japan; ^2^ Department of Medical Oncology Sapporo Medical University School of Medicine Hokkaido Japan

**Keywords:** colorectal cancer, colorectal neoplasm, endoscopic submucosal dissection, hyaluronic acid, sodium alginate

## Abstract

**Objectives:**

The usefulness of 0.6% sodium alginate (SA) as a submucosal (SM) injection solution for endoscopic SM dissection (ESD) has gained attention over the past few years. However, using ESD for colorectal neoplastic lesions is not explicitly researched as yet. Thus, we conducted this study to determine the feasibility and safety of 0.6% SA solution for colorectal ESD.

**Methods:**

In this single‐center, retrospective pilot study, a total of 100 cases treated with ESD using 0.6% SA as a SM injection solution for colorectal neoplasia at our institute were retrospectively reviewed to clarify the clinical feasibility and safety of 0.6% SA. The primary endpoint was to evaluate the complication rate, and the secondary endpoint was to determine the procedure time and the amount of solution used.

**Results:**

Intraoperative perforation was observed in 1 case (1.0%), 2 cases (2.0%) presented with postprocedural hemorrhage, and no lethal adverse events were observed. The median ESD procedure times were 39.5 min (10–150), and the amount of solution used was less than 20 mL in 67 cases (67.0%). En‐bloc resection could be achieved in 97 cases (97.0%). Although six cases underwent subsequent surgery due to the deep SM invasion (>1000 μm), there were no cases with nodal involvement, confirmed through histopathological evaluation.

**Conclusions:**

Our findings indicate that 0.6% SA can potentially ensure safe and secure ESD for colorectal neoplasia.

## INTRODUCTION

Colorectal cancer ranks third in incidence and second in mortality worldwide, accounting for 1.9 million new cases and 935,000 deaths in 2020.^1^ Despite therapeutic advances in patients with colorectal cancer, the outcomes are still poor, especially in the advanced stages.^2^ Therefore, early detection and therapeutic endoscopic intervention for colorectal neoplasia are undoubtedly important.

With the introduction of endoscopic submucosal (SM) dissection (ESD), the endoscopic resection of superficial colorectal neoplasia has recently shown remarkable advances. En‐bloc resection can be achieved with ESD, even in large lesions greater than 20 mm in diameter, contributing to a lower local recurrence than piecemeal endoscopic mucosal resection.^3^ Patients who underwent ESD with completely resected lesions report no metastatic occurrence within 5 years postoperatively.^4^

Currently, 0.4% sodium hyaluronate (SH) is regarded as one of the most reliable and widely used solutions for ESD for colorectal neoplasia.^5^ However, perturbation of high‐molecular‐weight SH reportedly contributes to cancer aggressiveness via Hippo signaling activation, as confirmed through molecular biology research.^6^ SH may stimulate the growth of residual tumor cells after endoscopic procedures such as ESD, followed by an upregulation of CD44 in murine models.^7^ Therefore, although 0.4% SH is a well‐recognized and suitable SM injection solution for colorectal ESD, careful follow‐up is required, particularly in cases with inadequate margins. Thus, a safer solution for SM injection is warranted for ESD.

Sodium alginate (SA) was extracted from algae for the first time in 1883 and has been used as a viscosity‐enhancing stabilizer and coagulator for food.^8^, ^9^ SA has been used as a digestive mucosal protective agent at a concentration of 5% for over 60 years in Japan (Alloid G; Kaigen Pharma).^10^ Thus, the efficacy and safety of SA are well established. Taking advantage of its viscosity, SA has been used as a SM injection solution for ESD.^11^, ^12^, ^13^ A 0.6% SA solution (Liftal K; Kaigen Pharma Co.) for SM injection was approved in Japan in 2018. Although a randomized control study revealed that the efficacy rate of SM injection using 0.6% SA was non‐inferior to 0.4% SH in ESD for esophageal and gastric neoplastic lesions, the feasibility and safety of 0.6% SA in ESD for colorectal neoplastic lesions have not yet been clarified.^14^ Those backgrounds inspired us to conduct a single‐center retrospective pilot study to evaluate the feasibility and safety of 0.6% SA solution for colorectal ESD.

## METHODS

### Cases

We retrospectively reviewed 100 colorectal neoplasms treated with ESD using 0.6% SA at our institute between October 2019 and September 2021. All cases with colorectal neoplasia treated with ESD using 0.6% SA during the period were included in this study. Informed consent was obtained from all the patients included in this study. This study was approved by the institutional review board at Otaru Ekisaikai Hospital (IRB 21‐07).

### ESD procedure

**FIGURE 1:**
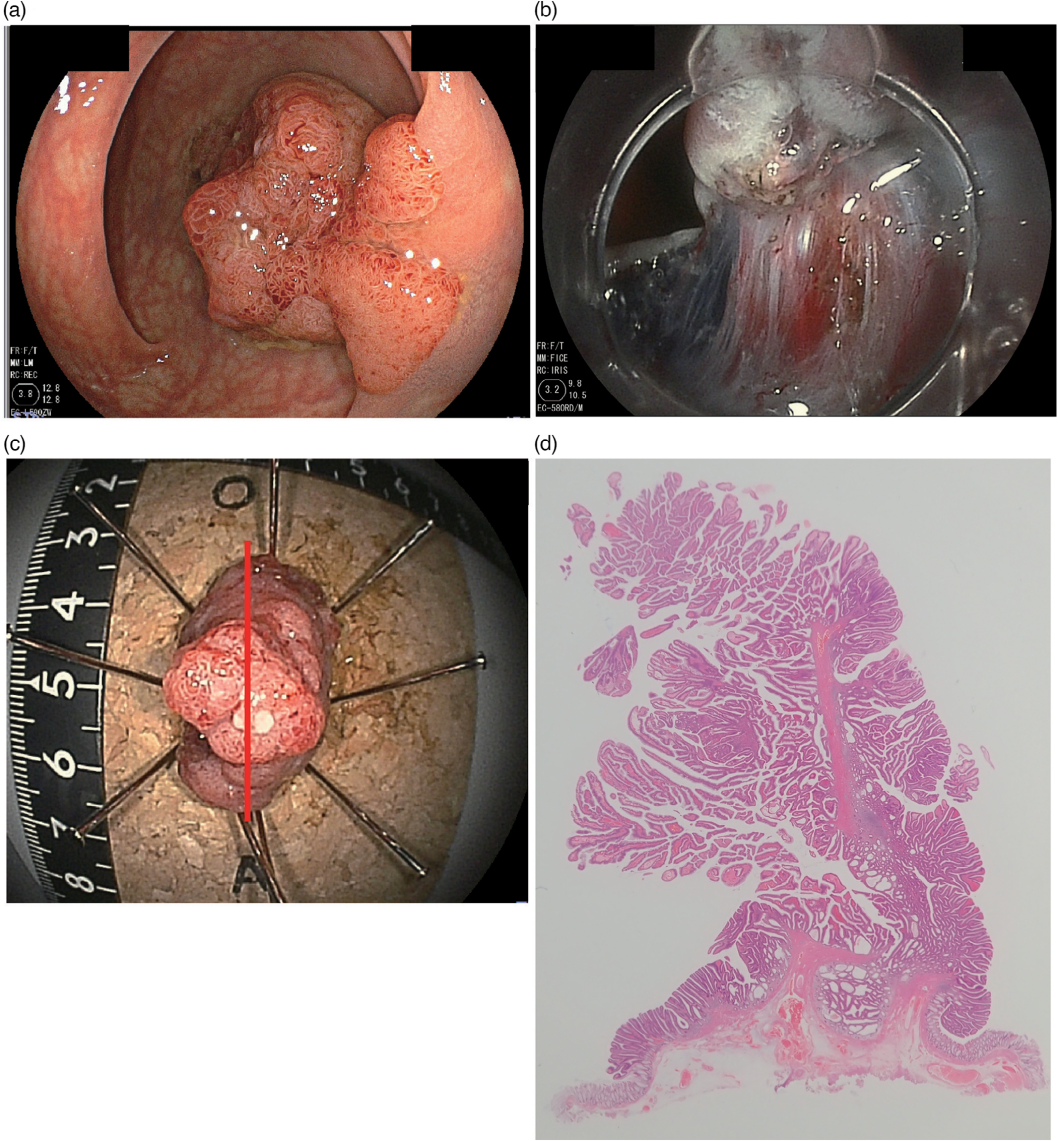
A case representation of one of the cases enrolled in the study: (a) the 30 mm sized 0‐Isp lesion was located in the sigmoid colon; (b) the endoscopic image during the submucosal dissection; (c) macroscopic view of the resected specimen; (d) the image of the lesion at the loupe statue (hematoxylin and eosin staining).

ESD was conducted by eight colonoscopists (two with more than 15 years of experience and 6 with less than 15 years of experience), using a double‐balloon endoscope (EI‐580BT or EI‐530B; FUJIFILM Co.) or an endoscope with a water jet (EC‐580RD; FUJIFILM Co.), depending on the location of the lesions and the colonoscopists’ choice. A solution of indigo carmine and epinephrine in glycerin–fructose (Glycerol; Taiyo Pharma Co., Ltd.) was first injected to lift the submucosa, and a mixed solution of indigo carmine and 0.6% SA was injected into the submucosa using a blunt needle (25 g, 3 mm in length, Top Corporation). Mucosal incision and SM dissection in all cases were conducted using a FlushKnife BT‐S (1.5 mm, FUJIFILM Co.) and electrosurgical generator (VIO 300D; Erbe Elektromedizin). One of the typical cases is represented in Figure 1.

### Study outcomes

As a pilot study, we evaluated the occurrence of adverse events, procedure time, and amount of injected solution treated with 0.6% SA. Perforation was defined as a hole visible during the procedure or free air detected with imaging tests, and post–procedural hemorrhage was defined as bleeding that required additional endoscopic treatment. The procedure time was defined as the time between the start of the mucosal incision and the completion of SM dissection.

## RESULTS

Table 1 presents the clinical characteristics of the cases in this study. The median age and sex ratio (male/female) of cases were 71.5 (42–90) years and 60/40. Preoperatively, 15 cases were treated using antithrombotic agents (mono antiplatelet therapy; 7 cases, mono anticoagulant therapy; 7 cases, combination of antiplatelet and anticoagulant; 1 case). We suspended antithrombotic treatment prior to ESD, if possible. In this study, however, ESD was conducted without cessation in three cases, considering the underlying condition. The median diameter of lesions was 20 mm (6–50), and 41 cases (41.0%) were found in the ascending colon, followed by the transverse colon (19 cases), sigmoid colon (14 cases), rectum (11 cases), descending colon (10 cases), and cecum (5 cases). The number of cases with laterally spreading tumor non‐granular type was 48 (48.0%), followed by laterally spreading granular tumor type (LST‐G; 34 cases) and elevated type (18 cases). Seven cases out of 100 converted to snare resection during the ESD procedure (hybrid ESD) because of technical difficulties.

**TABLE 1:** Characteristics of the patients included in the study.

Age (years)	
Median	71.5
Range	42–90
Sex, *n* (%)	
Male	60 (60.0)
Female	40 (40.0)
Antithrombotic agents intake, *n* (%)	15 (15.0)
Mono antiplatelet therapy	7 (7.0)
Mono anticoagulant therapy	7 (7.0)
Combination antiplatelet and anticoagulant therapy	1 (1.0)
Cessation of antithrombotic agent during ESD procedure	12 (12.0)
Size (mm)	
Median	20
Range	6–50
Location, *n* (%)	
Cecum	5 (5.0)
Ascending	41 (41.0)
Transverse	19 (19.0)
Descending	10 (10.0)
Sigmoid	14 (14.0)
Rectum	11 (11.0)
Classification, *n* (%)	
Elevated type	18 (18.0)
LST‐G	34 (34.0)
LST‐NG	48 (48.0)

Abbreviations: ESD, endoscopic submucosal dissection; LST‐NG, laterally spreading tumor non‐granular type; LST‐G, laterally spreading granular tumor type.

Table 2 shows the postoperative findings of the cases investigated in this study. The median diameter of the resected specimen was 25 mm (10–60). En‐bloc resection was performed in 97 cases (97.0%). Four cases resulted in an inconclusive horizontal margin on histopathological evaluation. There were no cases of lymphovascular invasion on histological evaluation. Furthermore, 6 cases (6.0%) underwent subsequent surgery due to the deep SM invasion (>1000 μm). Histological evaluation revealed no evidence of lymph node metastasis or residual tumor in all cases.

**TABLE 2:** Postoperative findings of the cases investigated in this study.

Size of resected specimen (mm)	
Median	25
Range	10–60
Conversion from ESD to hybrid ESD, *n* (%)	7 (7.0)
En‐bloc resection, *n* (%)	97 (97.0)
Negative resection margin, *n* (%)	
Horizontal margin	96 (96.0)
Vertical margin	100 (100.0)
Histological type, *n* (%)	
Tubular adenoma	41 (41.0)
Sessile serrated lesion	18 (18.0)
Tubular adenocarcinoma	38 (38.0)
pTis	26 (26.0)
pT1a	4 (4.0)
pT1b	8 (8.0)
Carcinoid	3 (3.0)
Others	0 (0.0)
Positive lymphovascular invasion, *n* (%)	
Lymphatic invasion	0 (0.0)
Vessel invasion	0 (0.0)
Subsequent surgery, *n* (%)	6 (6.0)
Lymph node metastasis, *n* (%)	0 (0.0)
Adverse events, *n* (%)	
Perforation	1 (1.0)
Postprocedural hemorrhage	2 (2.0)
Time (min)	
Median	39.5
Range	10–150
Injection volume, *n* (%)	
<20 mL	67 (67.0)
≥20 mL, <40 mL	27 (27.0)
≥40 mL	6 (6.0)

Abbreviation: ESD, endoscopic submucosal dissection.

Lethal adverse events were not observed in these cases. Intraoperative perforation was observed in 1 case (1.0%), and 2 cases (2.0%) presented with postprocedural hemorrhage, which required additional endoscopic treatment. The median procedure time was 39.5 min (10–150). Moreover, the volume of solution used was less than 20 mL in 67 cases (67.0%), 20–40 mL in 27 cases (27.0%), and more than 40 mL in 6 cases (6.0%).

## DISCUSSION

In this pilot study, we suggested the feasibility and safety of ESD for colorectal neoplasia using 0.6% SA as a SM injection solution. Few cases present adverse events such as perforation and postprocedural hemorrhage. Furthermore, the median procedure time was acceptable, and the total volume of solution used was less than 20 mL (one vial) in more than half of the cases contributing to cost reduction.

ESD for colorectal neoplasia is still considered a challenging procedure even for expert endoscopists in some situations, such as right‐sided location, large tumor size, and high degree of fibrosis.^15^ Several characteristics, such as the winding, many folds, and thinness of the colonic wall, also cause the technical difficulties of colorectal ESD.^16^ Indeed, the perforation rate related to colorectal ESD is reportedly as high as 1.4%–20.4%, which is higher than that associated with stomach ESD.^17^ Therefore, efficient SM elevation solutions are mandatory to maintain sufficient mucosal elevation and achieve safer ESD for colorectal neoplasia. In this study, the number of cases with perforation during the procedure was one (1.0%), and no cases presented post‐procedural perforation. Furthermore, the number of cases with bleeding was two (2.0%) that could be well manageable only with endoscopic hemostasis. The rates of cases achieved en‐bloc resection and negative resection margin were well acceptable (97 cases, 97.0%; and 96 cases, 96.0%; respectively). Based on these findings, SA should be considered an SM injection solution for colorectal ESD.

There are several limitations to this study. First, the data used in this study was obtained from a single center. Furthermore, the long‐term effects of 0.6% SA were not evaluated. As mentioned above, SH can potentially stimulate the growth of residual tumor cells after endoscopic procedures requiring close and long‐term follow‐up, particularly for patients with inadequate margins.^7^ In this regard, SA is likely to be safe and secure considering the ingredient, which needs further investigation.

In conclusion, our study revealed that 0.6% SA can potentially ensure a safe and secure ESD for colorectal neoplasia. Further investigations, including a high‐quality prospective study to evaluate its non‐inferiority compared to existing solutions such as 0.4% SH or a long‐term observational study to clarify the safety of 0.6% SA are warranted.

## CONFLICT OF INTEREST STATEMENT

Authors declare no conflicts of interests for this article.
